# Inactive hepatitis B carriers: outcomes of patients followed at Hôpital Principal de Dakar, Senegal

**DOI:** 10.11604/pamj.2018.31.49.16296

**Published:** 2018-09-20

**Authors:** Ibrahima Diallo, Bineta Ndiaye, Cheikh Abdoukhadre Fall, Mouminatou Mbaye, Imane Korch, Papa Silman Diawara, Ababacar Mbengue, Sara Boury Gning, Papa Saliou Mbaye, Fatou Fall

**Affiliations:** 1 Department of Internal Medicine and Hepato-Gastroenterology, Hôpital Principal de Dakar, Dakar, Senegal; 2 Department of Biology, Hôpital Principal de Dakar, Dakar, Senegal; 3 Department of Medical Imaging, Hôpital Principal de Dakar, Dakar, Senegal

**Keywords:** Chronic hepatitis B virus, Inactive carrier, evolutive profile, Senegal

## Abstract

The evolutive profile of inactive HBV carriers is variable. Patients can remain inactive, or may evolve into chronic active hepatitis or hepatocellular carcinoma. Aim: to describe the long-term outcome of chronic hepatitis B inactive carriers followed at Hôpital Principal de Dakar. This is a retrospective study including all inactive HBV carriers, followed since 2001, and with regular monitoring of at least 5 years. Transaminases, viral load and screening for hepatocellular carcinoma were performed every 6 to 12 months. We included 52 patients. The mean follow-up was 76.2 months (60-162), the mean age 36 years (13-62 years) and the sex ratio 0.93 (25 men, 27 women). Four patients (7.7%) had an ALT above the normal. Eleven patients (21.1%) had persistently elevated viral load greater than 2000 IU/ml, while in three cases (5.8%), this increase was transient. Twenty-six patients (50%) had a detectable viral load, but still below 2000 IU/ml. Twelve patients (23.1%) had an undetectable viral load for the duration of monitoring. Eleven patients (21.2%) underwent liver biopsy. The activity or fibrosis were minimal in all cases (A or F = 1) or absent (A or F = 0). Only four patients (7.7%), had HBs seroconversion after a follow-up of six, seven and ten years. There was no focal lesion or cirrhosis detected during the follow-up. After a follow-up of at least 5 years, inactive HBV carriers remain inactive in 92.3% of cases. Their evolutive profile is characterized by an absence of elevated liver enzymes but with fluctuations of the viral load. HBs seroconversion rate is low and the risk of progression to hepatocellular carcinoma almost nil.

## Introduction

Inactive hepatitis B virus (HBV) carrier state is defined according to European Association for the Study of the Liver (EASL) by chronic HBV infection evolves at least for 6 months, associated with normal ALT (Alanine aminotransferase), undetectable or very low serum HBV DNA levels below 2000 IU/ml, HBeAg negative, absence of viral coinfection C, D and HIV (human Immunodeficiency Virus) and a morphologically normal liver [1]. This diagnosis cannot be retained only after a regular follow of at least one year without changing these settings, due to possible fluctuations in transaminases and viral load. The evolving profile in the long term of these inactive carriers is variable, patients can remain inactive, or progress to chronic active hepatitis or hepatocellular carcinoma. This evolution has never been studied in Senegal, which is located in an endemic area. Our aim was to describe the outcomes of these patients followed at Hôpital Principal de Dakar for an inactive chronic carriage of HBV.

## Methods

It is a retrospective study including all patients followed since 2001 for an inactive HBV carrier state at Hôpital Principal de Dakar. Inclusion criteria were inactive HBV carrier (normal ALT, HBV viral load below 2000 IU/ml, HBeAg negative, absence of HCV, HDV or HIV co-infection, with a morphologically normal liver), and a follow-up of one year to confirm this diagnosis, and at least 4 years follow-up to 1 January 2015. Patients who have an inactive HBV carriage but with irregular follow-up or following less than 5 years since diagnosis, and those who had another cause of chronic liver disease, were not included. The data studied were: age at screening, sex, discovery circumstances of the portage of HBV, transaminases, ALP and GGT, bilirubin, TP, albumin, platelet count, alpha fetoprotein, hepatic imaging, serum HBV DNA. Patients had to have during their follow-up, clinical examination, dosage of transaminases and serum HBV DNA, and a screening of cirrhosis and hepatocellular carcinoma (HCC) all 6 to 12 months (alpha-fetoprotein and abdominal ultrasound). Viral reactivation was defined as a persistent increase of the viral load above 2000 IU/ml. All patients who had persistently elevated ALT or HBV DNA levels underwent a liver biopsy and/or an elastography (Fibroscan^®^) in search of a disease activity or significant fibrosis. The biopsy results were expressed according to the METAVIR score. The data were analyzed using SPSS 17 software.

## Results

**Figure 1:**
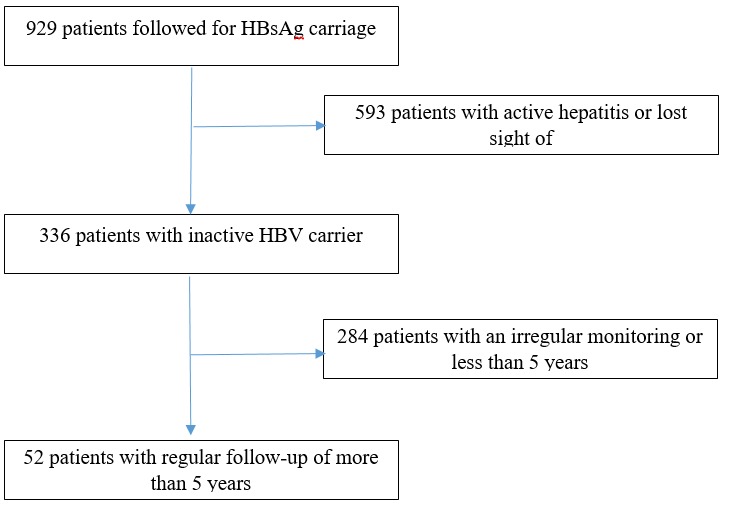
Patients selection

We included 52 patients among 959 people followed-up (Figure 1). The mean duration of follow-up was 76.2 months (60-162). There were 25 men (48%) and 27 women (52%). The mean age was 36 years with extremes of 13 and 62 years. The discovery circumstances of the portage of the virus were blood donation (59%), voluntary testing (22%) and oriented screening (19%). Clinical examination at the discovery of the infection and during follow-up were normal in all cases. There was no patient overweight or obese in our series. Patient characteristics at screening are summarized in Table 1. The anti-HBe was positive in all cases. Only 4 patients (7.7%) had ALT levels above normal, during follow-up, respectively in the 12^th^, 30^th^, 66^th^, and 72^th^ months. This elevation was isolated and transient in all cases, and among them only one had a concomitant increase of viral load. At the screening, the HBV viral load was undetectable in 17 patients (32.7%) and detectable in 35 patients (67.3%). During follow-up, viral load was constantly undetectable in 12 cases (23.1%) and detectable in 26 cases (50%) but still below 2000 IU/ml. Three patients (5.8%) had a transient increase of HBV DNA levels, and 11 (21.1%) had persistent elevation of viral load greater than 2000 IU/ ml. Among the latter, 6 had a viral load of less than 20 000 IU/ml, whereas for the other 5, it was permanently more than 20 000 IU/ml. Abdominal ultrasonography during follow-up was normal in 51 patients, while one patient had a suspicious lesion of HCC, but not confirmed by CT scan and MRI. Thus, no cases of HCC or cirrhosis was observed during the monitoring. Fibroscan was performed in 9 patients from those with elevated viral load and showed a score that remained below 7 kPa in 7 patients, and increased respectively to 9.9 and 10 kPa in 2 cases. A liver biopsy was performed in eleven patients (21.1%) who had persistently elevated HBV DNA levels above 2000 IU/ml. The activity or fibrosis according to the METAVIR score were minimal (A or F = 1) or absent (A or F = 0) in all cases. Only four patients (7.7%) lost HBsAg after six, seven and ten years of follow-up, with the apparition of anti-Hbs antibodies. There were two men and two women, aged when they were detected HBsAg positive 27, 53, 30 and 31 years. So after a follow-up of at least 5 years, 92.3% of inactive carriers of our series remained inactive and 7.7% had a HBs seroconversion.

**Table 1:** Patient characteristics

Settings	Mean	Extremes
Age (years)	36	13 - 62
Follow-up period (months)	76,2	60 - 162
ALT (UI/ml)	26	10 – 41
AST (UI/ml)	26	12 – 61
GGT (UI/ml)	29	5 – 70
ALP (UI/ml)	91	18 – 188
Total bilirubin (mg/l)	7,6	1 – 16
PT (%)	87	65 – 100
Albumin (g/l)	46	30 – 59
Hemoglobin (g/dl)	13,2	10 – 16,9
Platelets (/mm3)	246770	140000 – 383000
αFP	6,3	0,6 – 36

## Discussion

Hepatitis B can manifest in many forms with dynamic changes between the different phases. Inactive carriers represent more than two thirds of patients [2]. In a study at Hôpital Principal Dakar, 64% were inactive HBV carriers [3]. The outcome of these patients is rarely studied, particularly in Africa where we did not find any published study on this subject. This group has a favorable prognosis in the long term with a low risk of complications such as cirrhosis or hepatocellular carcinoma [1]. However, it is necessary to do regular monitoring every 6 to 12 months in these patients to detect these complications. We investigated the evolutionary profile on at least 5 years of these inactive HBV carriers in Senegal where 16 to 17% of blood donors are chronic carriers of HBsAg. However, the difficulties related to the cost and availability of examinations make our series quite short, only patients who had regular explorations as part of the monitoring, being included. Our patients are young subjects with a mean age of 36 years. This is probably related to the fact that our country has a high prevalence of HBV infection, this one being acquired in 90% of cases in infancy with perinatal transmission. All patients were asymptomatic at screening as well that during the monitoring. Liver function tests were normal, and during follow-up only 4 cases (7.7%) of transient ALT elevations were observed without aetiology found. In inactive HBV carriers, a transient increase in ALT without viral reactivation may affect up to one third of patients [4, 5]. Should be sought in this context others possible causes of cytolysis including drug intake, obesity, fatty liver, diabetes, factors found in the study of Tong [4]. The proportion of inactive carriers having an undetectable viral load is 34 to 45% [6-8]. Changes in viral load in these cases are variable during follow-up, which can remain below 2000 IU/mL, undetectable or not, or rise transiently or permanently. In our study, 21.1% of patients had persistently elevated viral load greater than 2000 IU/ml. This evolving mode of viral load was found in 21% of cases in the study of Tong [4], and 23.8% in a Greek population [9]. Thus, in inactive HBV carriers, the viral load remains below 2000 IU/ml during follow-up with or without transient fluctuations in 3 of 4 patients. A viral reactivation is possible in some patients especially in cases of spontaneous or induced immunosuppression, sometimes with a risk of fulminant hepatitis. None of our patients did not present an active hepatitis, ALT remained normal during follow-up. The reactivation rate is low with an annual incidence of 1.2 to 1.55% [5, 8]. It is higher in the endemic countries. The cumulative incidence is 17.1 and 21.6% respectively at 10 and 20 years [5]. Risk factors of spontaneous reactivation are an advanced age and male gender [5, 8, 10]. As part of monitoring, elastography (Fibroscan^®^), a non-invasive exam, easy to perform, is an important tool that can guide the realization of liver biopsy in some patients [1, 11].

We used it in 9 patients among the eleven who had elevated viral load, and their Fibroscan^®^ scores remained minimal (less than 7 kPa) in 7 patients and high in 2 cases. The Fibroscan^®^ score is low in inactive HBV carriers with a median of approximately 4.8 kPa [11]. Furthermore, progression of Fibroscan score of over 30%, was observed only in 2.8% of patients during follow-up, and this, especially in those with DNA levels greater than 20,000 IU/ml [7]. If in doubt about the occurrence of active hepatitis in inactive carriers, especially in front of persistently elevated ALT, high viral load or a significant Fibroscan^®^ score, liver biopsy can be performed. By against the realization of a liver biopsy is not necessary for diagnosis when the criteria of an inactive portage, apart from histology, are gathered. Indeed, histology is normal or with minimal lesions in 100% of inactive HBV carriers [12, 13]. Furthermore, the control to 4 years in liver histology of these inactive carriers, showed that the latter was unchanged in 97% of cases [14]. We conducted this examination in 11 of our patients who had a persistently high viral load even if there was not an increase in transaminases. And in all cases the fibrosis or inflammation was absent or minimal according to the METAVIR score. Thus, the realization of liver biopsy in inactive HBV carriers who present during follow-up normal ALT and viral replication between 2,000 and 20,000 IU/ml, is not necessary [15]. Elevated HBV DNA levels between 2000 and 20000 IU/ml and normal ALT levels do not mean disease activity [10]. In that case, Papatheodoridis, in his review of the literature suggests a quarterly monitoring of ALT and an annual dosage of HBV viral load and Fibroscan, and this for 2 years. If these parameters remain normal, switch to a biannual monitoring of ALT [15]. The occurrence of complications such as cirrhosis or HCC is rare in this population. The annual incidence of the occurrence of cirrhosis is estimated between 0.05% and 0.1% and is multiplied by 20 if there is a reactivation of the virus during evolution [5, 16]. Furthermore, the inactive HBV carriage is a risk factor for HCC, both for those with detectable DNA than those with undetectable viral load [6], with an annual incidence of 0.06% to 0.17% [4, 6]. Risk factors are age, male sex and alcohol consumption [6]. Monitoring of at least 5 years of our patients found no cirrhosis or HCC. Most studies reported rare cases of complications [4, 8, 9, 12]. Loss of HBsAg in inactive carriers is low with an annual incidence of 1.1 to 1.5% [4, 12, 17]. It occurred in 7.7% of our patients. It was, after 10 years, 8.7% in the series of Tohme in Alaska [8], 8.1% in the Taiwanese study of CHU [17]. Despite this seroclearance, patients should receive continuous monitoring. Even if HBsAg becomes negative, there is a possibility of persistent low detectable level of DNA at the origin of occult hepatitis [9, 18], and the risk of HCC, even if it is small, still persists, that anti-HBs antibodies are detectable or not [18, 19]. This risk is greater in patients with a seroclearance after 50 years [18].

## Conclusion

The inactive chronic HBV carriers in our study population in an endemic area, remain inactive in the long term. Their evolutive profile is characterized by an absence of cytolysis but with a fluctuation of HBV DNA levels and normal histology in those with persistent elevation of this one. The rate of loss of HBsAg is low and the risk of progression to hepatocellular carcinoma almost zero.

### What is known about this topic

- Inactive carriage of HBV rarely changes;
- There is a low risk of developing HCC in these patients, hence the importance of regular monitoring;
- There is also a possibility of Hbs seroconversion.

### What this study adds

- There is no data on the subject in sub-Saharan Africa;
- The minimal follow-up of 5 years in our patients makes it possible to have an idea of the evolution of hepatitis b in inactive carriers in sub-Saharan Africa.

## Competing interests

The authors declare no competing interests.
